# Understanding Rural Women’s Domestic Work Experiences (DWE) in Ibadan, Nigeria: Development of a Measurement Tool Using Confirmatory Factor Analysis

**DOI:** 10.3390/ijerph182111043

**Published:** 2021-10-21

**Authors:** Abisola Osinuga, Brandi Janssen, Nathan B Fethke, William T Story, John A Imaledo, Kelly K Baker

**Affiliations:** 1 Department of Occupational and Environmental Health, College of Public Health, University of Iowa, Iowa City, IA 52242, USA; Brandi-janssen@uiowa.edu (B.J.); Nathan-fethke@uiowa.edu (N.B.F.); kelly-k-baker@uiowa.edu (K.K.B.); 2 Department of Community and Behavioral Health, College of Public Health, University of Iowa, Iowa City, IA 52242, USA; william-story@uiowa.edu; 3 Department of Health Promotion and Education, University of Ibadan, Ibadan 200212, Nigeria; hisgracejohn@gmail.com

**Keywords:** domestic work, women, rural, water insecurity, factor analysis

## Abstract

Gender norms prescribe domestic labor as primarily a female’s responsibility in developing countries. Many domestic tasks depend on access to water, so the physical, emotional, and time demands of domestic labor may be exacerbated for women living in water-insecure environments. We developed a set of domestic work experience (DWE) measures tailored to work in rural areas in developing countries, assessed rural Nigerian women’s DWE, and examined relationships among the measures. Interviewer-administered survey data were collected between August and September from 256 women in four rural Nigerian communities. Latent factors of DWE were identified by analyzing survey items using confirmatory factor analysis. Pearson’s correlation was used to examine relationships among latent factor scores, and multivariate linear regression models were used to determine if factor scores significantly differed across socio-demographic characteristics. The DWE measures consisted of latent factors of the physical domain (frequency of common domestic tasks, water sourcing and carriage, experience of water scarcity), the psychosocial domain (stress appraisal and demand–control), and the social domain (social support). Significant correlations were observed among the latent factors within and across domains. Results revealed the importance of measuring rural Nigerian women’s DWE using multiple and contextual approaches rather than relying solely on one exposure measure. Multiple inter-related factors contributed to women’s DWE. Water insecurity exacerbated the physical and emotional demands of domestic labor DWE varied across age categories and pregnancy status among rural Nigerian women.

## 1. Introduction

Despite growing female participation rates in the workforce and the growing contribution of women to economic growth and gross domestic products (GDP) globally, the fact remains that women are still primarily responsible for domestic and care work [1]. Millions of women in rural Asia and Sub-Saharan Africa are estimated to spend 10–17 h per day performing domestic work [2,3]. In developing countries, domestic tasks entail engaging in demanding tasks such as cleaning, food preparation and cooking, water fetching, manual washing of dishes and clothes, child/elder care, and engaging in subsistence agriculture [4,5,6]. For example, in Gujarat, India, women spend between three to four hours of their day collecting only water [7]. Ownership of modern household appliances is often limited in rural sub-Saharan Africa. Thus, domestic tasks such as food preparation can be long, often strenuous, involving tasks such as water fetching, heavy lifting, pounding, grinding, and cooking for long hours [8]. Water insecurity further worsens the burden of domestic labor. Water infrastructure is poor, and access to water is unreliable in many rural communities in sub-Saharan Africa, including Nigeria. Nigeria had the second-highest proportion of women spending more than 30 min/day on water collection in a study analyzing the time spent on water collection labor across 24 Sub-Saharan African countries [9]. Since most domestic tasks depend upon water, the physical, emotional, and time demands of cooking and other domestic work may be exacerbated for water-insecure households in low-income countries (LIC). Performance of many domestic tasks for those who depend heavily on rainwater storage and rainfall-recharged wells in rural Nigeria can become problematic during drought periods [9,10]. The responsibility for procuring water in these water-insecure environments rests on women who spend much time and physical effort on water collection [9,11].

Beyond economic and geographic influences, socio-cultural norms and gender ideologies impact household division of labor and women’s domestic work experiences. The traditional gender role theory described by Parson and Bales (1955) delineated the paid work domain and instrumentality as important for men, with the home domain and expressiveness as integral for women [12]. This theory on societal perception of domestic responsibilities is still relevant today in 21st-century Nigeria. Akanle and Ejiade (2012) aptly described gender roles in a traditional Nigerian society [13]. In Nigeria, performance of domestic labor is viewed as the ‘woman’s domain’ regardless of women’s engagement in paid employment outside of the household [14]. In Yoruba Nigerian culture (the primary study population in this paper), a housewife with long leisure time is considered lazy. Additionally, a housewife’s sense of pride and accomplishment is often linked to her resourcefulness and ‘busyness’ within the home [15]. Likewise, a man performing normative roles may be perceived as feminine (e.g., cooking) or called a ‘women wrapper’ in traditional, rural Nigeria society [16]. Thus, socio-cultural norms may influence women’s domestic experiences, their bargaining power over the time allocated to domestic labor, and male involvement in domestic responsibilities [17].

Domestic labor and water insecurities have mainly been studied as separate issues affecting women’s physical and psychosocial health. However, water carriage, which is a consequence of water insecurity [18], and high domestic work burden are associated with musculoskeletal pain (MSP) among women [19]. As a result, evidence is still lacking on how these risk factors are interrelated, specifically on how the experiences of water insecurity could influence the performance of tasks and women’s domestic work experiences (DWE). Measures commonly used to define DWE include: the number of working hours [20,21,22,23,24], the level of familial support with domestic responsibilities [25,26,27], the biomechanical demands of domestic tasks [5,28,29,30], perceived stress and demands of work [31], and the frequency of task performance per week or self-reported ‘intensity-ratings’ of specific domestic tasks [32,33,34]. Identifying the interdependencies between these DWE measures, including other measures such as water insecurity and water carriage, could improve understanding of rural women’s DWE and related health outcomes.

Furthermore, most studies on women’s DWE originated in developed countries and considered the synergistic effects or consequences of paid and unpaid work demands [35,36,37]. DWE was assessed using ‘time’/‘frequency’ measures in these studies. DWE influence on physical and psychosocial health was secondarily evaluated, based on their capacity to confound the association between the risk factors from paid work and health or contribute to psychosocial strain from poor work-life balance among women residing in primarily urban environments [17,32,36,38].

The consequence for engaging in domestic work can be far-reaching, beyond time lost. It influences women’s capacity to pursue productive economic activities, increasing physical and psychosocial stress, especially for those balancing domestic care work with paid employment [25,39,40,41]. Unpaid care work has been associated with school absenteeism among girls, thus perpetuating the cycle of poverty and gender inequality. Understanding women’s domestic work realities and the consequences of such work on women’s health will be crucial towards developing interventions focused on improving the redistribution of work responsibilities between men and women [41]. 

### The Domestic Work Experience Model

**Figure 1:**
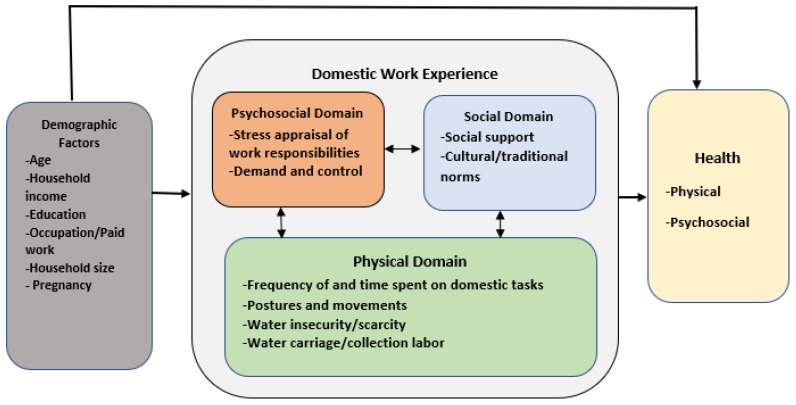
The Domestic Work Experience Model.

The purpose of this study was to develop DWE measures tailored to the context of work in rural areas of LICs, such as Nigeria, and assess women’s DWE. Domestic tasks are mostly performed manually, and water insecurity (which may increase physical stress) is prevalent in these environments. We hypothesize that physical, psychosocial, and social factors of living and working conditions all contribute to women’s DWE, and that DWE varies across socio-demographic variable categories (Figure 1). These DWE, in turn, could impact women’s physical and mental health (Yellow Box, Figure 1).

Physical Factors [Green Box, Figure 1]: Long hours spent on domestic work, including the difficulty and frequency of domestic tasks, contribute to DWE by leading to work overload, increased time pressure, reduced opportunities for rest and recovery, and increased perceived stress [21,35]. Furthermore, the performance of tasks in extreme postures, water insecurity, fetching, and carriage activities could influence the efficiency of task performance, contributing to DWE and physical stress.

Psychosocial Factors [Orange Box, Figure 1]: Psychosocial stress results from the interaction between external demands, such as work responsibilities, and the cognitive appraisal of the capacity to cope with those demands [42]. The transactional model of stress theory posits that stress appraisal may influence stress reactivity, including coping strategies used to manage stressful situations. Likewise, women’s perception of domestic work responsibilities as stressful or not (cognitive appraisal) contributes to their DWE [43,44]. The demand–control model of occupational psychosocial stress posits that psychological job demands and decision latitude (level of autonomy or agency over work) influence perceived stress and negative stress reactivity [45,46]. Women who perform time-demanding domestic tasks over which they have low control/agency may experience psychological distress [17,47,48]. 

Social Factors [Blue box, Figure 1]: Social support and networks influence women’s capacity to adapt to stressful domestic work conditions and mitigate the adverse effect of strenuous domestic work burden on their physical and mental health [22,26,27,49]. Low social support or social networks reduces opportunities for responsibility-sharing and division of labor [50,51], while greater social support, especially instrumental support (division of domestic labor), could improve women’s DWE and health [27].

Demographic Factors [Grey Box, Figure 1]: Situational and contextual determinants of health, such as socioeconomic status or life stage and pregnancy status, may moderate women’s DWE, including the exposure and reaction to domestic work stressors [49,52,53]. Although variability in levels of domestic work stress/exposure is not the focus of this study, the model proposes that the magnitude of domestic work exposure varies over time, life stage, season, and place. 

We hypothesized that there would be interrelatedness within and across the physical, psychosocial, and social factors of DWE and that sociodemographic characteristics would moderate DWE. In this study, we used confirmatory factor analysis (CFA) to test our hypotheses about the relationships among items representing the physical, psychosocial, and social domains of DWE for rural, low-income Nigerian women. This approach allowed us to identify the latent factors of women’s DWE, the extent of factor interrelatedness, and the factors that most influence DWE, including the direction of the inter-relationships.

## 2. Methods

### 2.1. Study Setting

A cross-sectional study was designed to measure factors that contribute to DWE among women in rural Ibadan, Nigeria. Ibadan, the capital of Oyo state, is one of the largest cities in Africa in terms of landmass and one of the most densely populated Nigerian cities. The projected population by the year 2025 in the city is 5.03 million people [54]. There are 11 local governments in Ibadan, five in the metropolitan urban area, and the rest primarily made up of semi-urban/peri-urban and rural settlements. Increased population growth and rural–urban migration has led to the formation of peri-urban, semi-rural slums on the outskirts of Ibadan metropolis. These fringe communities live in the reality of abject infrastructural deprivation. For example, at least 60% of rural and semi-rural households in Oyo State, Nigeria, must fetch water from streams, rivers, and unprotected wells to meet their water supply needs [55]. We recruited 365 women of reproductive age (i.e., 18–49 years) from four neighboring rural communities in Lagelu and Akinyele Local Government Areas, Oyo State, Nigeria. These communities represented typical communities where cultural and gender norms promote domestic responsibilities as ‘women’s work’ and where household water access is low [13,55]. 

### 2.2. Survey Design

Item Development: A literature review was conducted between February and March 2019 to identify items from validated surveys for consideration in a DWE instrument. Items included those related to two primary areas: physical work and psychosocial work. Physical work included experience of water insecurity [56]; water sourcing and proximity to water source from the Joint Monitoring Program (JMP) core questions [57]; load carrying [58]; and posture and movements [29,59,60]. Psychosocial work included items related to decision authority and psychosocial job demands from the Job Content Questionnaire [61]. Other items related to physical work—the frequency of domestic work and water fetching and carriage—and psychosocial work—perceived difficulty of performing domestic tasks—were also developed. A third primary area was also developed related to social support. A questionnaire consisting of new and modified items from validated surveys was created. 

Cognitive Interviewing, Pretest, and Survey Administration: After the questions were developed, cognitive interviews were carried out among ten women to assess whether participants interpreted the meaning of the questions as intended and to determine if the response options were appropriate [62]. Modifications were made on ambiguous and culturally irrelevant items. The revised survey was subsequently pretested among 40 women of similar demographic characteristics as the study participants. The undeclared pretest (respondents not aware it was a pretest) was conducted to ascertain whether respondents were interpreting questions correctly, question variation, difficulty, flow, order, and time spent per question. After the pretest, modifications were made on ten questions related to physical work, water insecurity, and water carriage.

### 2.3. Measures

Socio-Demographic Factors: The sociodemographic factors included in this study were age (18–25, 26–30, 31–35, 36 years and above), income (lowest quartile, median, and highest quartile), education (primary, secondary, and tertiary), household size (0–3 people, 4–6 people, >6 people), age of youngest child (above or below five years), youngest child is walking (yes or no), pregnancy status (yes or no), occupation (no paid work, semi-skilled labor, and skilled labor), and hours of paid work per week (continuous, but categorized using quartiles for analysis purposes). 

Physical Factors of DWE:

- Frequency of common domestic work: Participants were asked to select how frequently they perform each of 14 common domestics tasks using a 5-point Likert scale (never/not me, rarely (three times/month), sometimes (two or three times/week), every day, more than once a day).
- Time (h/week) on domestic tasks: Response entry for the total time spent on domestic tasks per week was categorized into three groups: lowest third (<23 h/week), middle (23–30 h/week), and the highest third (>30 h/week).
- Lifting and Loading: Six items were used to measure ‘water fetching and carriage practices’ during domestic tasks. These items included: responsibility for water collection labor (only me, me and others, others only); number of water trips per collection period recoded into four ordered categories (None, <5 trips, 5–8 trips, >8 trips); quantity of water carried per trip (None, <25 L, 25–30 L, >30 L); where carried water was placed (head loading, on hands, use of assistive device); and how water containers to be carried were lifted (no lifting, assisted, unassisted lifting). Apart from water containers, the frequency of carrying children while performing household tasks (never–always), where children are placed on the body (on the back, hip, arm), and the frequency of lifting other loads (>25 pounds) per week were assessed.
- Proximity to Water Source: Three items were used to measure water-source proximity. They included: ‘time taken to reach water collection point’ (none, ≤5 min walk, 6–10 min walk, >10 min walk), ‘time taken to complete a water collection trip’ (not applicable, <10 min, 10–20 min, >20 min), and ‘where water source/collection point is located’ (inside dwelling, around the compound, elsewhere).
- Experience of Water Insecurity: Three items were adapted and modified from the household water insecurity scale [56]. Participants were asked to rate how frequently they were worried, angry, or frustrated that they did not have enough water, or rationed water usage in homes in the past four weeks on a 5-point Likert scale (never to always).

Psychosocial Factors of DWE:

- Psychosocial Appraisal of Domestic Work Stress: Six items were used to measure respondents’ appraisal of domestic work. Three items each measured women’s positive and negative cognitive appraisal of domestic work responsibilities on a 5-point Likert scale (strongly agree to strongly disagree).
- Time Demand–Control: Two item-statements (freedom to choose when and how to perform tasks) were modified from the ‘decision authority’ and one item-statement (time pressure) was modified from the ’psychological job demand’ subscales of the Job Content Questionnaire (JCQ) [61], constructed on a 5-point Likert scale (strongly agree to strongly disagree).

Social Factor of DWE:

- Social Support: Three items measuring how frequently respondents ask for or receive assistance from household members were constructed on a 5-point Likert scale (never to always). All the variables included as measures were all potential independent variables that could be utilized in predicting health or mental wellbeing outcomes. The complete survey tool can be found in (Supplementary Materials File S1).

### 2.4. Study Design and Data Collection

The finalized survey containing the above items and demographic questions was administered by interview to study participants in the four selected communities. Data were collected in August and September 2019 from women residing in households in selected communities. The interviewers were six graduate students from the State University who were fluent in the local language and experienced at carrying out community surveys. Two interviewers were designated to each community. Interviewers approached prospective participants from every fifth house on streets in the communities, introduced the research, ascertained the woman’s eligibility by asking three questions (age, engagement in domestic work, and presence of any chronic illness), and obtained informed consent before proceeding with survey administration. Of the 365 women who agreed to participate in the study, questionnaire data were collected from 356 women, yielding a completion rate of 98%. Missing values in all variables of interest did not exceed 4%. Examination of missingness using Little’s MCAR test (Little, 1998) was not significant (χ^2^ *p*-value = 0.19). 

### 2.5. Ethics

The study met the required regulatory requirements for the protection of human research participants. The study was approved by the Ethics Review Committee of the Oyo State Ministry of Health, Planning, Research, and Statistics department (approval ID:AD/13/479/223) and by the University of Iowa Institutional Review Board (IRB ID: 201904718). Before the recruitment of study participants, the community heads and village heads were approached for support. Informed consent was obtained from all participants. Women not engaged in domestic work, women over the age of 49 years (reproductive ages), and those with chronic illness and disabilities were excluded. 

### 2.6. Analytic Strategy

Descriptive and Preliminary Analyses: Categorical variables were summarized using frequencies and percentages. Normally distributed continuous variables were summarized using means and standard deviations. Non-normally distributed continuous variables were summarized using medians and interquartile ranges. Normality assumptions were examined among continuous variables measuring DWE. Polychloric (inter-item) and polyserial (item–total) correlations were also estimated to assess the interrelationships among variables in the DWE domains. 

Data Screening and Management: The Kaiser–Meyer–Olkin (KMO) measure of sampling adequacy [63,64] and the Bartlett’s test of sphericity were used to assess the adequacy of performing factor analysis on the data [65]. The criterion for an acceptable KMO estimate was <0.70. Variables with individual measures of sampling adequacy <0.50 were excluded from factor analysis for each item. The Bartlett’s test (*p* < 0.05) of sphericity was used to examine if the correlation between items was greater than expected by chance. 

Other criteria used to determine the a priori exclusion of items from the final confirmatory factor analysis (CFA) model included (1) >20% missing responses, (2) insignificant factor loadings <0.35, (3) items with negative error variance, (4) high cross-loading, (5) low communalities (R^2^ < 0.40) per item, (6) weak inter-item and item–total correlation (<0.35), and (7) high influence on reduction in Cronbach’s alpha coefficient. Reliability was determined using Cronbach’s alpha <0.7 and the mean inter-item and item–total correlation <0.35 as acceptable cut-offs for each measure of DWE [66]. 

Confirmatory Factor Analysis: CFA uses item–factor relationships and model fit indices to evaluate whether the covariance matrix of the observed data matches the covariance matrix of a hypothesized model. Based on previous literature and the conceptual model created, CFA models were used to assess the inter-relationships among the DWE measures (observed variables) and their latent constructs. 

Since all items were categorical (ordinal data), a robust weighted least square estimation with mean and variance adjustment (WLSMV) was used to estimate model parameters—the residual variances among items, and covariance between factors, and the estimated factor loadings. The goodness-of-fit for CFA models was determined by the comparative fit index (CFI), the Tucker–Lewis index (TLI), the root mean square error of approximation (RMSEA), and the Kline method for assessing model fit using the χ^2^ statistic. As a rule of thumb, CFA and TLI values of ≥0.9 signifies very good model fit. For RMSEA, values of ≤0.06 indicate good model fit, values ≤0.08 indicate moderate model fit, and values ≥0.1 indicate poor fit [67]. Lastly, a non-significant χ^2^ or a ratio of χ^2^ to degree of freedom (df) <3 demonstrate a good model fit for the Kline method [68]. The cut-off value used to determine item loading on a related factor was 0.40 with *p*-value < 0.05. Modification indices were added to improve model fit while the factor structure essentially remained the same. Factors were allowed to correlate, and the variance of each factor was set to 1.0 in all models.

Convergent validity was assessed by examining the strength of the average variance extracted (AVE > 0.5) of each construct, i.e., the average R^2^ per factor was >0.5. Construct-level discriminant validity was ascertained if the squareroot of AVE for each construct was greater than the correlations of the construct with other constructs. Raw mean scores (per factor) were generated and presented by demographic variables. Standardized regression-based factor scores were estimated for all observations by multiplying the inverse of the item’s correlation matrix value by the correlation matrix value of its factor loadings. All analysis, including CFA analysis was performed using the Lavaan and semPaths package in R [69].

Pearson’s correlation was used to examine the relationship among latent factor scores of DWE (within and across domains). Multiple linear regression models (one model per latent factor) were used to examine the extent to which each regression-based factor score of DWE changed across the categories of sociodemographic variables. Statistical significance was determined at an alpha level of 0.05.

## 3. Results

### 3.1. Participant Characteristics

More than half (62%) of the participants reported having started or completed secondary school education. Most (84%) were employed in semi-skilled, informal labor such as petty trading, tailoring, and hairdressing. The mean age was 30.8 years (SD = 6.5) and median monthly household income was NGN 15,000/USD 40. Approximately half lived in households ranging from four to six members. A tenth of the women was pregnant (11.2%), half had children under five years of age, and more than half (66%) relied on dug wells for water supply. Head loading was the most prevalent (68%) means of transporting water carriage. Women spent an average of 29 h/week and 41 h/week on domestic and paid work, respectively. 

### 3.2. Internal Consistency and Adequacy of DWE Items for Factor Analysis

After evaluating item suitability for factor analysis, 21 items were dropped during preliminary analysis. Four items relating to the ‘frequency of common domestic tasks’ (grinding, pounding food, gardening, fetching firewood) were dropped because of low variability in participants’ responses. Twelve items relating to ‘posture and movements’ during domestic tasks were dropped because of high missing responses and low variability in participants’ responses. For example, most participants (260) indicated sitting as usual posture even for tasks that typically do not require sitting, such as sweeping and washing clothes. Three items measuring ‘positive appraisal of domestic work’ were dropped because they had low Cronbach alpha, mean, and item–total correlation. Internal consistency for the remaining 31 items of DWE measure were then re-assessed using the Cronbach’s alpha. All items demonstrated good sub-scale reliability, except for ‘stress-appraisal’ and ‘demand/control constructs’ (α = 0.68 and 0.67) (see Supplemental Table S1). The Kaiser–Meyer–Olkin measure of sampling adequacy was 0.71, which is above the recommended cutoff of 0.6, and the Bartlett’s sphericity test was significant (χ^2^(378) = 3283.07, *p* < 0.001). All these value indicators suggested that all 31 items could be used in our CFA models. 

### 3.3. Confirmatory Factor Analysis

**Figure 2:**
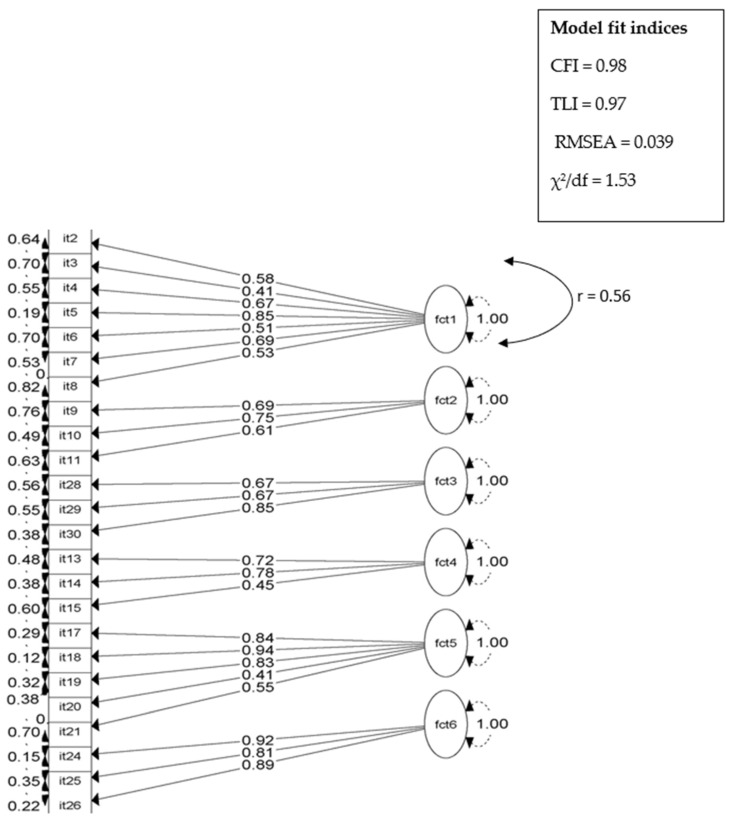
Six-factor model of DWE showing the final confirmatory factor analysis model. ‘It’are variable items included in the final model, 2–8 (Factor 1, Frequency of domestic tasks); 9–11 (Factor 2, Stress appraisal); 13–15 (Factor 3, Demand and control); 17–21 (Factor 4, Water sourcing, and carriage); 24–26 (Factor 5, Experience of water scarcity; 28–30 (Factor 6, Social support). Values to the left of the numbered items are error variances; values within the lines are factor loadings of each observed variable on the latent factors; 1.00 indicates that the variance of each factor was set to 1; r = correlation specified between Factor 5 and 6.

Standardized parameter estimates (factor loadings, robust standard errors, variances, and R^2^) from each item are presented in Table 1. All six measures of DWE demonstrate strong theoretical fit. The factor loadings were all significant *(p* < 0.001) and higher than the set cut-off (>0.4). The AVEs of each construct were all higher than the acceptable value of 0.40 and greater than the correlation coefficients among factors, which shows strong discriminant validity. The goodness-of-fit statistics of the three CFA models are presented in Table 2. First, a model consisting of seven factors and 30 items was tested. Items with negative variances (items 23 and 31), low communalities or R^2^ (items 1, 12, 16, 22, and 27), high cross-loadings with other items (items 1 and 31) and low factor loadings (item 1) were excluded from the model. The remaining 24 items shared at least 40% of their variance with their designated factor (R^2^ ≥ 0.4), indicating adequate convergent validity. Next, a second model consisting of seven factors and 24 items was tested. Finally, a third model of six factors (combined proximity to water source and water carriage into one factor, because of high inter-factor correlation) was tested in which three correlation terms were included between highly correlating items (items 7 and 8, 17 and 18, 19 and 21) from the same factor. All models met the criteria for an acceptable model as all fit indices were well within the recommended cut-offs. However, the third model had the best fit (CFI = 0.98; TLI = 0.97; RMSEA; χ^2^/df =1.5) and was selected as the final model. The final DWE measures consisted of: physical factors, which included 16 items across three latent factors (frequency of domestic tasks, water sourcing and carriage, and experience of water scarcity); psychosocial factors, which included six items across two latent factors (stress appraisal and demand and control); and social factors, which included three items in one latent factor (social support) (see Table 1 for final items by factor; Figure 2 for CFA Path Diagram; see Supplemental Table S2 for the DWE tool).

### 3.4. Description of Items in the Physical Domain

Frequency of Domestic Work (never to always): The most frequent common domestic tasks women engaged in every day or more than once a day were cooking (93%), bathing and dressing children (90%), sweeping (87%), washing dishes (90%), fetching water (75%), carrying children (75%), and washing clothes (64%). Most women reported almost never or rarely engaging in fetching and carrying firewood (75%), pounding food (79%), grinding food (74%), or gardening/planting (76%). The mean score for all items in the factor was 3.07, meaning most women engaged in many of the listed tasks every day/sometimes (two or three times/week). Respondents ranked sweeping (63%), washing dishes (21%), and cooking (20%) as the top-three easiest domestic tasks and ranked fetching and carrying water (45%), manually washing clothes (29%), and cleaning toilets and bathroom (24%) as their top-three most physically strenuous tasks.

Water Fetching and Carriage: Approximately 25% of the women had on-plot water services within their household, 21% had a water source (mostly well source) located around their compound, and the remaining half had a water source located outside their dwelling/compound (53%). About one-quarter (23%) did not have to fetch water (0 min walking distance), 25% walked around their compound to access water (≤5 min walking distance), 38% spent between 6 and 10 min, and 15% spent more than 10 min. About 22% did not have to complete a water trip, 24% of the women spent less than 10 min completing a water trip from their compound to their household, 35% spent 10–20 min, and 19% spent more than 20 min completing a water trip. Regarding the number of water trips, approximately 21% did not have to complete a water trip. Another 21% completed fewer than five trips per collection period, 46% completed between five and eight water trips per collection period, and 12% completed more than eight trips per collection period. 

Experience of Water Scarcity (never to always): Almost half of the respondents reported that they never experienced water scarcity consistently across the three items. The remaining half reported experiencing some form of scarcity, whether rarely, occasionally, or often. Women reported feeling worried often (10%), sometimes (20%), and rarely (25%) about having enough water in the past 30 days. Women reported rationing water often (6%), sometimes (21%), and rarely (31%) in the past 30 days. Lastly, women reported feeling angry/frustrated often (12%), sometimes (21%), and rarely (21%) about not having enough water to complete domestic tasks in the past 30 days. 

### 3.5. Description of Items in the Psychosocial Domain

Stress Appraisal (Strongly Agree to Strongly Disagree): Of the four stress appraisal statements, women most often responded “strongly agree” or “agree” to: “Felt drained after completing domestic tasks” (28%); “Doing household tasks requires a lot of physical effort” (40%); and “Caring for children requires a lot of physical effort” (35%).

Demand and Control (Strongly Agree to Strongly Disagree): Of the four demand and control statements, women most often responded “strongly agree” to: “Have adequate time to complete domestic tasks for the day” (51%); “Have adequate time for hobbies and other meaningful activities” (44%); and “I can choose not to do domestic work when tired or exhausted” (46%).

### 3.6. Description of Items in the Social Domain

Social Support (Never to Always and Yes/No): A total of 64% of the women reported that they ask for assistance from their family members. Women then rated their frequency of asking for and receiving assistance from family members. They mostly responded “always” (68%) or “often” (33%) to ‘asking for assistance with domestic tasks from family members’ and mostly responded ‘always’ (33%) or ‘often’ (32%) to “getting help with housework from family members”.

### 3.7. Relationship among the Latent Variables of DWE

Within-Domain Relationships: Within the physical domain of DWE, high frequency of domestic work scores was significantly correlated with increased experience of water scarcity (r = 0.31; *p* < 0.01), but not with water sourcing and carriage (r = 0.10; *p* > 0.15) (Supplemental Table S3). Increased water sourcing and carriage scores were associated with an increased experience of water scarcity (r = 0.20; *p* < 0.01). In the psychosocial domain, increased stress appraisal was significantly associated with increased demand and control (high demand and low control) scores.

Across-Domain Relationships: High frequency of domestic work scores were significantly associated with increased stress appraisal (*r* = 0.36; *p* < 0.01) but decreased social support (*r* = −0.25; *p* < 0.01) and demand and control (*r* = −0.32; *p* < 0.01). Water sourcing and carriage had non-significant relationships with stress appraisal and demand and control, but a high score was correlated with decreased social support (*r* = −0.17; *p* < 0.01). Experience of water scarcity was positively associated with stress appraisal (*r* = 0.13; *p* < 0.01), but had no significant relationships with demand and control and social support. Increased stress appraisal (*r* = 0.17; *p* < 0.01) and increased demand and control (*r* = 0.14; *p* < 0.01) were associated with decreased social support.

### 3.8. Relationship between the Latent Variables of DWE and Demographic Factors

Women’s Age: Women in older reproductive age groups (36 years and above; 31 to 35 years) had significantly lower (*p* < 0.05) demand and control and higher water sourcing and carriage scores (Table 3) when compared to women in the youngest reproductive age group (18 to 25 years). Women in older reproductive age groups (31 years and above) had higher social support scores (*p* < 0.01). There was no significant difference in frequency of domestic work, experience of water scarcity, or stress appraisal scores by women’s age.

Pregnancy Status: Women who were pregnant had significantly higher demand and control scores but lower frequency of domestic tasks scores (*p* < 0.05) when compared with non-pregnant women. There was no significant difference in water sourcing and carriage, experience of water scarcity, stress appraisal, or social support scores by pregnancy status.

Household Income: Lower household income was significantly associated with higher (*p* <0.01) frequency of domestic tasks, water sourcing and carriage, and social support, but lower demand and control scores. There was no difference in experience of water scarcity or stress appraisal by income level.

Level of Education: Low (primary or no formal education) education was significantly associated with higher mean and regression-based water sourcing and carriage scores. There was no significant association between level of education and other DWE measures.

Household Population: Increased household size population (more than six people) was significantly associated with lower mean and regression-based frequency of domestic tasks and demand and control scores, but higher social support scores. 

Child’s Age and Walking Status: Having a child under five years of age was not associated with scores from any DWE measures but having a child who was not walking yet was significantly associated with increased frequency of domestic tasks (*p* < 0.01) and experience of water scarcity (*p* < 0.05).

## 4. Discussion

This study documented DWE conditions among rural Nigerian women and developed a measurement tool that accounted for the psychometric properties of, and relationships among, measures of DWE. Rather than focusing upon one or two DWE indicators, we developed the measures using established behavioral, work psychology, and gender theories. We tested our hypothesized framework of relationships using CFA by analyzing the internal consistency and predictive, convergent, and discriminant validity. Results indicate that the final CFA model was robust, and the latent factors derived were reliable and valid measures of DWE of women in rural Nigeria. This new measurement is important because there are no standardized or validated indicators to assess domestic work burden or experiences among women in the literature [31]. 

The DWE measures reflect three main domains: women’s interaction with their physical environment and the physical aspect of domestic work (physical domain); psychosocial appraisal of domestic work responsibilities (psychosocial domain); and how social support influences work burdens (social domain). When examining the relationships within each domain, there were significant inter-relationships within the physical domain. Timely management and performance of daily domestic work responsibilities, such as manual laundry, cooking, and childcare, depended on women’s access to water, which is supported by prior research [70]. Thus, water insecurity, water carriage, and domestic work are not separate entities as they had been addressed in the prior literature. Women were primarily responsible for performing multiple domestic tasks every day [71], and they ranked manual laundry—a water-dependent task—and water-fetching/carriage as the most difficult tasks. This result reinforces the economic and health benefit of investing in water-supply technologies over the past decades and the need to increase the number of households with access to improved water sources.

Certain physical-domain factors also varied across sociodemographic characteristics. Frequency of engaging in domestic tasks significantly differed across levels of household income, household size, pregnancy status, and childcare-giving status. Low-income, non-pregnant women from large households and those taking care of young children (<12 months) most frequently engaged in domestic tasks. The practice of water carriage decreased as women advanced in age but increased among low-income, less-educated women. This result could be because children support their mothers, assisting with domestic responsibilities as both the women and children get older. The level of a woman’s education was directly related to her paid and household income. Education predicted the capacity to acquire wealth-based assets such as on-plot water infrastructure that can reduce domestic work demands.

Within the physical domain, women’s experience of water scarcity was also related to increased water carriage and sourcing for water outside of homes. In this study, women’s experience of water scarcity was lower than expected. Increased population density in Ibadan metropolis has influenced the rate of home ownership in rural areas. In the last 20 years, urban-to-rural migration of middle-income families has increased because of ease of land and home ownership [72]. This phenomenon of urban–rural migration may have influenced the sociodemographic characteristics, DWE, and coping strategies among women in these rural areas. Many women live in abject poverty (as evident from the average household income), and few have on-plot water infrastructure. Nonetheless, few women experience water scarcity or rely on an unimproved water supply because of the communal water-sharing strategies adopted in the environment. Communal and household water sharing have been identified as adaptive buffers used to deal with water insecurity in LICs [55,73,74]. 

There were also inter-relationships within the psychosocial domain; women with high stress appraisal scores also had high demand and control scores. Women who perceived their domestic work to be stressful (stress appraisal) also reported that they were time-pressured, did not have time for leisure activities, and had less control over the completion of tasks (demand and control). Psychosocial stress results from the interaction between external demands, such as domestic work responsibilities, and women’s internal cognitive appraisal or perception of their capacity to cope with work demands. Demand and control significantly differed across age categories, levels of household income, and household size. In agreement with previous studies, demands of work—including perceived control and frequent engagement in domestic tasks—significantly reduced among high-income, older women and those living in larger households (more than six people) [47,49,53]. Living with limited resources and without mechanical domestic appliances or public-provided utilities to lessen domestic work burden, women often rely on support from their grown children [75]. The reverse was observed among pregnant women, whose perceived demand and stress appraisal increased despite the reduced frequency of engaging in domestic tasks. It is possible that stressors from paid work, in addition to pregnancy-induced stress, may have influenced their perceived domestic work demands [44]. 

For the social domain, spousal and children’s assistance with domestic work responsibilities (social support) increased as women aged, as income increased, and as household size increased. Strong social support systems have been found to positively influence a myriad of women’s health outcomes [76] in the literature. These include improved psychological resilience to violence [77], food insecurity [78], and water insecurity [74,79]; improved access to maternal health services [70]; improved psychosocial health [20]; and improved musculoskeletal health [50,51]. Thus, poor social support may reduce the opportunities for responsibility-sharing and division of labor. 

### Strengths and Limitations

This study took a multi-factorial approach to systematically measure women’s DWE in rural Nigeria and provided the groundwork for further research into the inter-relationships between factors contributing to the DWE in similar populations. However, some of the items in the tool may not apply to the context of work in other populations. These measures need to be validated and contextualized among other populations. Future research in other populations could pilot and adapt this survey tool (Supplementary Materials File S1) using this same approach to identify relevant items and measures for that context, which would provide more information on the validity of these measures across populations. 

The tool presented in this study mainly focused on the negative experience of work and was limited in capturing the coping strategies or resilience mechanisms women utilize to cope with domestic work. This tool’s test–retest reliability analysis was not performed, which could have given more information on the stability of the relationships between the measures over time and the ability to account for seasonal variation in DWE. Some items from the final CFA model were removed because of poor internal consistency, low unique and negative variances, and low or cross-factor loadings. Question rewording and ordering, and further validation studies will be required to vigorously assess if the items contribute to the latent factors of DWE measures.

Furthermore, self-report biomechanical risk factors of domestic work could not be accurately assessed, such as awkward postures, repetitive movement, bending, and squatting from respondents. A systematic review found that the ability of respondents to correctly recall and assess their posture and movement using questionnaires across studies was low [80]. Posture and movement items probably had low variation in responses because of a low level of literacy or lack of understanding of the response options. As a result of this, our DWE measure may not have fully captured the physical factors/demand of domestic work. Another limitation was that our cross-sectional design measured a snapshot of women’s DWE at one time. Since variations in exposures and DWE can be time, season, and life-stage dependent, the study design, and by extension the tool, may be limited in determining with precision the physical and psychosocial demands of domestic work. Future studies need to take a longitudinal, mixed-methods approach in assessing and understanding women’s DWE as well as combine the strengths of other methods (observation and instrument-based methods) in quantifying the physical demands of domestic work. 

## 5. Conclusions

This study demonstrated that multiple factors contribute to rural women’s DWE, as evident from the inter-relationships among measures across and between domains. This study revealed the importance of taking a multi-factorial approach when measuring rural women’s DWE, rather than relying solely on frequency/time measures. Water insecurity and carriage particularly contributed to and influenced women’s DWE because they were linked to increased work demands, lack of social support, and decision authority over task completion. Engaging in demanding domestic labor is the occupational reality of many rural Nigerian women. Using standardized and contextually appropriate tools and measures such as the DWE in understanding and quantifying the impacts of domestic labor, relevant behavioral, infrastructural, and ergonomic interventions can be developed to advocate for and reduce the physical and mental burdens from domestic labor. This tool can be used to evaluate what aspect of women’s domestic labor and experiences contribute most to adverse health outcomes (e.g., musculoskeletal or psychosocial health outcomes). The measure can also help recognize and advocate for potential areas of influence that should be targeted to achieve gender equality. For example, the measures can be incorporated in the gender-specific sustainable development goals (SDG) indicators targeting gender inequality (those assessing gender gaps in unpaid care/domestic work). Finally, the measure will be useful in developing appropriate interventions that can reduce the burden of domestic work on women. 

## Figures and Tables

**Table 1:** Parameter estimates and factor loadings from confirmatory factor analysis, Model 1.

DWE Constructs	Observed Variable	β	SE	Variance	R-Square
Frequency of domestic tasks α_total_: 0.75 AVE:0.63 Mean ± SD: 2.96 ± 0.76	1. Fetching water **	0.30	0.079	0.90	0.10
	2. Sweeping	0.75	0.063	0.47	0.54
	3. Cleaning	0.41	0.065	0.80	0.60
	4. Carrying children	0.72	0.077	0.50	0.44
	5. Bathing and dressing children	0.69	0.068	0.40	0.88
	6. Washing clothes	0.41	0.072	0.86	0.45
	7. Cooking meals	0.71	0.069	0.72	0.47
	8. Washing dishes	0.52	0.081	0.82	0.51
Stress appraisal α_total_: 0.69 AVE:0.61 Mean ± SD:2.04 ± 0.77	9. Feel drained or tired after completing domestic tasks for the day	0.50	0.064	0.75	0.76
	10. Doing household tasks requires a lot of physical energy	0.86	0.079	0.47	0.52
	11. Caring for children requires a lot of physical energy	0.63	0.068	0.61	0.48
	12. Taking care of the home is not stressful **	0.42	0.084	0.35	0.15
Demand and Control α_total_: 0.73 AVE: 0.56 Mean ± SD:1.82 ± 0.86	13. Adequate time to complete domestic tasks	0.74	0.081	0.42	0.58
	14. Adequate time for hobbies or other meaningful activities	0.76	0.85	0.43	0.56
	15. Can choose not to do domestic work when tired	0.43	0.092	0.82	0.41
	16. Enjoyed doing domestic work **	0.35	0.101	0.79	0.18
Water Sourcing and Carriage α_total_: 0.83 AVE: 0.65 Mean ± SD: 2.34 ± 0.93	17. Distance to water source	0.89	0.021	0.60	0.75
	18. Where is source located	0.86	0.019	0.71	0.89
	19. Time to complete water trip	0.82	0.025	0.41	0.59
	20. Number of trips	0.45	0.048	0.83	0.42
	21. Quantity per trip	0.55	0.047	0.70	0.63
	22. Type of loading **	0.35	0.076	0.88	0.23
Experience of Water Scarcity α_total_: 0.9 AVE:0.55 Mean ± SD:1.96 ± 0.98	24. Worry about having enough water	0.91	0.017	0.42	0.85
	25. Rationed water usage	0.80	0.024	0.47	0.65
	26. Angry or frustrated about not having enough water	0.85	0.022	0.51	0.78
	27. Did not have enough water for household activities **	0.53	0.043	0.71	0.29
Social Support α_total_: 0.77 AVE: 0.51 Mean ± SD: 2.06 ± 0.91	28. Receive assistance (frequency)	0.664	0.045	0.56	0.43
	29. Ask for assistance from family members	0.84	0.053	0.42	0.72
	30. Ask for assistance (frequency)	0.675	0.051	0.54	0.46

Standardized factor loadings: SE = standard error; α_total_ = average Cronbach’s alpha per construct; AVE = Average variance extracted after items are excluded; ** items are excluded from final model.

**Table 2:** Final confirmatory factor analysis models and their fit indices.

Model	Parameters (N)	χ^2^	df	χ^2^/df	CFI	TLI	RMSEA (95% CI)	SMR
Model 1	115	601	303	1.98	0.94	0.93	0.053 (0.046–0.059)	0.081
Model 2	105	376	230	1.63	0.97	0.96	0.042 (0.035–0.05)	0.068
Model 3	97	292	191	1.53	0.98	0.97	0.039 (0.029–0.047)	0.067

CFI = comparative fit index, TLI = Tucker–Lewis Index, RMSEA = root mean square error of approximation.

**Table 3:** Factor scores from domestic work experience measures across socio-demographic variables.

Sociodemographic Characteristics	Physical Factors			Psychosocial Factors		Social Factor
	Frequency of Domestic Work	Water Sourcing and Carriage	Experience of Water Scarcity	Stress Appraisal	Demand and Control	Social Support
All	2.96 (0.76)	2.34 (0.93)	1.96 (0.98)	2.04 (0.77)	1.82 (0.86)	2.06 (0.91)
Age						
18–25 years ^#^	2.89 (0.75)	2.51 (0.85)	1.94 (1.02)	2.02 (0.78)	2.06 (0.93)	1.81 (0.85)
26–30 years	2.94 (0.73)	2.33 (0.86) *	1.99 (1.06)	2.07 (0.76)	1.78 (0.82) **	2.00 (0.86) *
31–35 years	3.00 (0.79)	2.18 (0.95) **	1.83 (1.00)	2.03 (0.77)	1.71 (0.85) *	2.13 (0.81) **
36 years and above	2.95 (0.77)	2.14 (1.03) **	2.06 (0.97)	2.02 (0.79)	1.73 (0.79) *	2.33 (0.87) **
Pregnancy Status						
Non-pregnant ^#^	2.99 (0.74)	2.32 (0.93)	1.97 (1.02)	2.03 (0.77)	1.78 (0.85)	2.05 (0.90)
Pregnant	2.74 (0.88) *	2.31(0.97)	1.90 (0.98)	2.07 (0.79)	2.12 (0.92) *	2.08 (0.91)
Income/Month						
Highest Third ^#^	2.84 (0.79)	2.09 (0.96)	1.86 (0.98)	2.04 (0.78)	2.05 (0.91)	2.3 (0.86)
Median Third	3.00 (0.72) **	2.37 (0.91) **	2.02 (1.03)	2.01 (0.76)	1.63 (0.79) **	2.1 (0.94)
Lowest Third	3.04 (0.73) **	2.59 (0.80) **	2.02 (1.03)	2.01 (0.81)	1.77 (0.83) **	1.9 (0.93) *
Education						
Tertiary Education ^#^	2.92 (0.74)	1.97 (0.94)	1.93 (1.01)	2.93 (1.01)	2.09 (1.01)	2.13 (1.04)
Secondary	2.95 0.77)	2.40 (0.92) **	1.94 (1.02)	1.93 (0.95)	1.93 (1.01)	2.04 (0.91)
Primary Education	3.03 (0.70)	2.50 (0.88) **	2.09 (1.00)	2.01 (0.83)	1.93 (1.00)	2.01 (0.91)
Household Size						
0–3 people ^#^	2.88 (0.8)	2.29 (0.88)	1.93 (1.05)	2.05 (0.79)	2.08 (0.9)	1.87 (0.87)
4–6 people	2.97 (0.74)	2.32 (0.93)	1.95 (1.01)	2.06 (0.76)	1.78 (0.86) **	1.94 (0.88)
More than 6 people	2.87 (0.76) **	2.33 (0.98)	1.98 (0.89)	1.99 (0.79)	1.70 (0.81) **	2.37 (0.81) **
Child Walking						
Yes ^#^	2.92 (0.78)	2.30 (0.94)	1.89 (0.98)	2.02 (0.76)	1.76 (0.83)	2.09 (0.89)
No	3.16 (0.70) **	2.33 (0.92)	2.08 (1.03) *	2.05 (0.79)	1.85 (0.86)	2.00 (0.93)
Age-range of Child						
Over 5 ^#^	3.01 (0.75)	2.37 (0.93)	2.00 (0.99)	2.01 (0.79)	1.74 (0.89)	2.10 (0.93)
Under 5	2.91 (0.76)	2.26 (0.94)	1.92 (1.04)	2.07 (0.76)	1.90 (0.82)	2.02 (0.87)

Numbers are mean (SD); * *p* < 0.05; ** *p* < 0.001. ^#^ Indicates reference categories for sociodemographic variables.

## Data Availability

The data presented in this study are available on request from the corresponding author. The data are not publicly available due to privacy and ethical reasons.

## Supplementary Materials

The following are available online at https://www.mdpi.com/article/10.3390/ijerph182111043/s1. Table S1: Descriptive and reliability analysis of the DWE Measures. Table S2: The final domestic work experience survey questions. Table S3: Correlation between and across DWE regression-based factor scores. File S1: The Domestic Work Experience Questionnaire (Original Tool).
