# Supplementary material for: Understanding Rural Women’s Domestic Work Experiences (DWE) in Ibadan, Nigeria: Development of a Measurement Tool Using Confirmatory Factor Analysis
Source: Int J Environ Res Public Health. 2021 Oct 21;18(21):11043. doi: 10.3390/ijerph182111043 (PMC8582860; doi:10.3390/ijerph182111043)
Supplement: Supplementary file 1 [file ijerph-18-11043-s001.zip › ijerph-1379706-supplementary.pdf]

Table S1. Descriptive and reliability analysis of the DWE measures

DWE Measures	Mean	SD	IIT	ITC	Cronbach's Alpha
**Frequency of Common Tasks (*Never to Always*)**					**α^{total} = 0.72**
1. Fetch water	2.79	0.91	0.25	0.27	0.73
2. Sweeping	3.19	0.66	0.25	0.30	0.72
3. Cleaning	2.20	1.00	0.23	0.42	0.71
4. Carrying Children	2.85	0.96	0.21	0.59	0.70
5. Bathing and Dressing Children	3.23	0.70	0.19	0.76	0.66
6. Washing Clothes	2.65	0.68	0.24	0.36	0.74
7. Cooking meals	3.33	0.68	0.20	0.69	0.67
8. Washing Dishes	3.15	0.70	0.24	0.55	0.72
**Stress Appraisal (*Strongly Agree to Strongly Disagree*)**					**α^{total} = 0.67**
9. Felt drained or tired after completing domestic tasks	2.03	0.73	0.38	0.46	0.65
10. Doing household tasks requires a lot of physical energy	2.04	0.77	0.25	0.77	0.62
11. Caring for children requires a lot of physical energy	2.04	0.82	0.29	0.65	0.63
12. Taking care of the home is not stressful.	1.81	0.72	0.43	0.40	0.69
**Demand and Control (*Strongly Agree to Strongly Disagree*)**					**α^{total} = 0.68**
13. Adequate time to complete the domestic tasks.	1.80	0.89	0.34	0.51	0.64
14. Adequate time for hobbies or other meaningful activities.	1.83	0.83	0.24	0.52	0.76
15. Enjoy doing domestic work	1.32	0.67	0.59	0.25	0.74
16. Can choose not to do chores when tired	1.83	0.71	0.30	0.50	0.58
**Proximity to water Source**					**α^{total} = 0.9**
17. Distance to water source	2.50	0.93	0.80	0.82	0.89
18. Where is source located	2.78	0.86	0.69	0.91	0.81
19. Time to complete water trip	2.70	1.01	0.78	0.83	0.88
**Water Carriage**					**α^{total} = 0.83**
20. Number of Trips/per collection period	2.35	0.91	0.54	0.72	0.78
21. Quantity per trip/collection period	2.48	0.95	0.60	0.69	0.82
22. Type of Loading	2.57	0.75	0.44	0.92	0.70
23. Support while lifting water	2.39	0.74	0.63	0.64	0.84
**Experience of Water Scarcity**					**α^{total} = 0.86**
24. Worry about having enough water for all needs. (Frequency)	2.01	1.05	0.54	0.87	0.78

25. Rationed water usage (Frequency)	1.89	0.92	0.52	0.87	0.76
26. Angry or frustrated about not having enough water (Frequency).	1.97	1.07	0.57	0.83	0.80
27. Did not have enough water for household activities (Yes/No)	1.27	0.44	0.76	0.60	0.90
**Support**					**α^{total} = 0.77**
28. Get assistance from family members (frequency)	2.45	1.02	0.65	0.57	0.73
29. Ask assistance from family members (Yes/No)	1.64	0.48	0.66	0.56	0.72
30. Ask assistance from family members (frequency)	2.45	1.19	0.76	0.45	0.62
31. Who is Responsible for water fetching	1.64	0.74	0.33	0.30	0.77

SD= Standard Deviation; IIT= Mean Inter-item correlation; ITC= Item-total correlation, α^{total} = average Cronbach's alpha per construct.

Table S2. The final Domestic work experience survey questions

DWE Measures	Response 1	Response 2	Response 3	Response 4	Response 5
**Frequency of Common Domestic Tasks**	Never/not me	Rarely	Sometimes	Everyday	More than Once a day
1. Fetching and Carrying water					
2. Sweeping the floor					
3. Cleaning					
4. Carrying Children					
5. Bathing and dressing for Children					
6. Manually washing clothes					
7. Cooking Meals					
8. Washing Dishes/kitchen ware					
**Water Sourcing and Carriage**					
17. Distance to water source	None	<5 minutes' walk	6 -10 minutes' walk	11 minute and above	
18. Location of water source	Inside Dwelling	Within the Compound	Elsewhere		
19. Time to complete a round water trip	None	< 10 minutes	10 -20 minutes	>20 minutes	
20. Number of Water trips	None	< five trips	5-8 trips	>8 trips	
21. Quantity of water carried per trip			5-9		
**Experience of Water Scarcity**	Never	Rarely	Sometimes	Often	Always
24. Worry about having sufficient water for household needs					
25. Rationed water usage because of scarcity or difficulty in collecting water					
26. Angry or frustrated about not having sufficient water					
**Stress Appraisal**	Strongly Disagree	Disagree	Neutral	Agree	Strongly Agree
9. Felt drained or tired after completing domestic tasks for the day					
10. Doing household tasks require a lot of physical energy					

11. Caring for children requires a lot of physical energy					
**Demand and Control**	Strongly Agree	Agree	Neutral	Disagree	Strongly Disagree
13. Adequate time to complete domestic tasks assigned for the day					
14. Adequate time for hobbies and other meaningful activities					
15. Can choose not to do domestic work when tired or exhausted					
**Social Support**	Never	Rarely	Sometimes	Often	Always
28. Do you ask family members for assistance with domestic tasks (Yes/No)					
29. How often do you get assistance from family members					
30. To what extent does family/spouse/child help with domestic work					

Table S3. Correlation between and across DWE regression based-factor scores

	Physical A	Physical B	Physical C	Psycho A	Psycho B	Social
Frequency of Domestic Tasks	1.00	0.10<br>$p = 0.15$	0.31<br>p <0.001	-0.359<br>$p < 0.001$	-0.32<br>$p < 0.005$	0.25<br>$p < 0.001$
Water Sourcing and Carriage		1.00	0.16,<br>$p = 0.002$	0.08<br>$p = 0.13$	-0.07<br>$p = 0.17$	-0.168<br>$p = 0.001$
Experience of Water Scarcity			1.00	0.15<br>$p = 0.012$	-0.065<br>p = 0.256	-0.08<br>$p = 0.41$
Stress Appraisal				1.00	0.18<br>$p = 0.0014$	0.17<br>$p = 0.0014$
Demand/Control					1.00	0.14<br>$p = 0.007$
Social						

File S1

_DOMESTIC WORK EXPERIENCE QUESTIONNAIRE (ORIGINAL TOOL)

INSTRUCTION

- We are researchers from the University of Iowa College of Public Health. We are interested in understanding how you perceive performance of domestic work and what coping resources you use to deal with stress.
- You are chosen to complete this survey because you are a woman between 18-49 years.
- Please respond to the questions from the Interviewer after indicating your consent.
- There are no right or wrong answers, so please answer the questions as they best relate to you.
- If you choose to respond to these questions, please feel free to provide genuine and accurate answers.
- If you do not fully understand or clearly hear the questions asked by the interviewer, please feel free to ask the interview to repeat herself.
- Completing this survey is completely voluntary, so please signify to the interviewer if you do not wish to provide an answer to a question or if you wish to stop at any time. Your responses will remain confidential and will not be linked back to you.
- The interview should take between 25-30 minutes to complete.
- When you have completed this questionnaire, the interviewer will give you a gift item.

SECTION A: SOCIO-DEMOGRAPHIC CHARACTERISTICS

1. Age ____________(years)

2. Weight ________________________ (kg) Height _________________(cm)

3. **Marital status** of respondent

☐Single
☐Live with married spouse at home
☐ Does not live with married spouse at home
☐Widowed
☐ Divorced/separated
☐Others ______________________ (**please specify**)

4. How many **people** live in household? _________ **(number**)

5.

6. How many **children** live in household?

☐ Below 5 years ______
☐ Below 14 years _______

☐ Others ______________________ ***(please specify, number of children and age)***

7. Highest level of **education** of respondent

☐ No formal education

☐ Primary School not completed

☐ Primary School completed

☐ Secondary School not completed

☐ Secondary School completed

☐ Tertiary education

☐ Others ______________ ______________ (please specify)

8. Ethnicity

☐Yoruba

☐Hausa

☐Igbo

☐Others ____(Please specify)

9. Religion of respondent

☐Christianity

☐Islam

☐Traditional

☐Others ______________________________(Please specify)

10. Pregnancy status of respondent

☐Pregnant

☐Non-pregnant but have been in the past (***please skip to question12***)

☐Never being pregnant **(*please skip to question 19*)**

11. If pregnant, how **old** is your pregnancy? i.e. (**months of pregnancy)** _____________ (**Weeks of pregnancy**) ________

12. Is this your first pregnancy?

☐Yes (***skip to question 22)***

☐No

☐ Yes ____ **(what are they)**

13. Please **fill** in the information about **child/children** (from youngest to oldest child). Fill in **"W"** if child is walking and **"NW"** if child is not walking yet.

**Demographics of Child**	**Child One (under 5)**	**Child Two (under 5)**
Age of Child (years /months)		
Weight of Child ***(RA weigh child if child is present)***		
Child Walking (W)		
Child Not Walking (NW)		

SECTION B: WATER ACCESS, SOURCING AND COLLECTION PRACTICES

Access to water: Question 19 to 28

14. What is/are the main source(s) of water regularly used by you and your household for drinking, washing, cooking and other purposes?

Mark TWO answer choices at MOST.

☐ Piped water into house/compound (running tap water present in houses or compound),
☐ Public tap/standpipe (fetch water from neighbor's tap or public taps)
☐ Protected dug well (get water from well-covered well)
☐ Unprotected dug well (get water from not cased, uncovered well)
☐ Rainwater collection
☐ Surface water (river, dam, lake, ponds, stream, canals)
☐ Other (Please specify) ______________

15. What are the **alternate** sources of water used by your household for drinking, washing, cooking and other purposes?
Mark ONE answer choice at MOST.

☐ Piped water into house/compound (running tap water present in houses or compound),
☐ Public tap/standpipe (fetch water from neighbor's tap or public taps)
☐ Protected dug well (get water from well-covered well)
☐ Unprotected dug well (get water from not cased, uncovered well)
☐ Rainwater collection
☐ Surface water (river, dam, lake, ponds, stream, canals)
☐ Other (Please specify) ______________

16. How would you rate the availability of water at your water source in the past two weeks?
☐Rarely (once in a while)
☐Occasionally (some of the time)
☐Frequently (almost all the time)
☐Always (every time)
17. Where is the **main** water source located?
☐ Inside your dwelling
☐ Around your compound (outside your dwelling) own yard/plot
☐ Anywhere else (please specify) _____________

18. **If elsewhere**, how long does it take you to get to your water point, get water and come back home?
Provide the time in minutes or hours.
____________ (minutes) (hours)
☐I do not know/cannot estimate

Water collection labor: Question 19 to 26
19. **Who** usually goes to the source to fetch the water **used by your household**? (**select all that apply**)
☐Me
☐Adult woman
☐Adult man
☐Female child _______ (age)
☐Male child ___________ (age)
☐Others ________________ (please specify)
☐I do not know

20. **What** is the typical distance covered when you go to fetch water (***Prompt respondent to describe how far they walk***)?
☐ Not applicable (water in homes)
☐ Not more than few steps
☐ Opposite my house
☐ 2-5 minutes walking distance (within same street)
☐ 6-10 minutes walking distance
☐ 11-20 minutes walking distance
☐ >20 minutes walking distance
☐ Others ___________________(specify)

21. **How** would you describe your **terrain/pathway** of walk from water point?
☐ Normal
☐ Hilly
☐ Uneven
☐ Muddy
☐ Bushy
☐ Other__________________(please specify)

22. How **many trips** do you usually make to the water collection point? ________ (**trips**)
☐ I do not know/cannot estimate
☐Not applicable (**woman** ***is not responsible for water collection***)

23. What is the **quantity of water** you carry per trip to water collection point___________ (**liters**)?
(***you can tell respondents to show you the bucket/container they use to fetch water and describe the type of bowl if you cannot estimate in litres, the popular bath bowls are 30-35 litres)***
☐I don't know
☐Not applicable (**woman** ***is not responsible for water collection***)

24. How much **water on average,** do you **use daily** as a household (one bucket is assumed to contain 20 liters) _____________________ ________________________

(***ask them if they finish the water in their 120litre drum every day, or ask how many water bowls they fetch and is finished, estimate from there***)
☐Cannot Quantify

25. How do you **carry** water?
☐on my head
☐wheelbarrow
☐in my hands
☐on my shoulder
☐Not applicable
☐Others____________ (Please Specify)

26. How do you **lift or lower** your bowl of water after fetching?
☐ unassisted
☐ assisted by another
☐ support with knee,

☐ support on well
☐ other

Question 27 to 29: Experience of water scarcity

27. In the past month, **has there been any time** when your household **did not have enough** quantity of water to use for household activities (drinking, cooking, bathing, washing) when needed?

☐Yes
☐ No

28. If yes to question (23) (***ask the respondents***), What do you think is the reason for this?

____

29. Please mark an "X" below the response options that most closely describes your experience with water sourcing and **usage in the past 30 days**.

**Questions**	**Never**	**Rarely (1-2 times/month)**	**Sometimes (3-10 times/month)**	**Often (more than 10 times/month)**	**Others _______(please specify)**
How often did you worry about your household having enough water for all its needs?					
How often did you or any household members use less water than you needed because there was not enough water or because it was too difficult to collect more water?					
How often did you feel angry or frustrated about not having enough water for the household? ***(Meaning any water at all, whether for watering crops or livestock, washing your hands, washing clothes, or any other needs)***					

SECTION C: DOMESTIC WORK HISTORY AND PRACTICES

30. Do you make money from doing any work? (***Paid work is defined as a woman earning cash/kind from her job or business***)

☐Yes
☐No

31. If yes, what are the works/business that gives you money? (***Probe the woman to list all types of work that they do that fetches them money, a woman can have more than once source of income)***

____ (please specify)

32. How many hours per day do you spend on your business/paid jobs per day for the past two weeks?
(Respondents may be best able to describe how many hours they spend per day, the two weeks provide a representative time frame for the respondent to remember their typical work schedule)

____

33. How many hours and time range do you usually spend on **doing chores within the home each day of the week?**

Monday ______ (hours /time range in morning) _____ (hours/time range in evening)
Tuesday ______ (hours /time range in morning) _____ (hours/time range in evening)
Wednesday ______ (hours /time range in morning) _____ (hours/time range in evening)
Thursday ______ (hours /time range in morning) _____ (hours/time range in evening)
Friday ______ (hours /time range in morning) _____ (hours/time range in evening)
Saturday ______ (hours /time range in morning) _____ (hours/time range in evening)
Sunday ______ (hours /time range in morning) _____ (hours/time range in evening)

Question 34: frequency of Common Domestic Tasks

34. Please mark an **"X"** below the response options selecting how frequently you perform the tasks.

**Domestic Task**	**Never/ not me**	**Rarely (3 times/month)**	**Sometimes (2 -3 times/week)**	**Everyday**	**Twice in the day**	**Others _______ (specify time)**
Sweeping the floor						
Mopping the floor						
Cleaning the toilet and Bathroom						

Fetching and carrying water
Fetching and carrying firewood
Carrying food produce
Washing dishes/kitchen ware
Manually washing clothes
Grinding food manually
Pounding food (mortar &pestle)
Cooking
Bathing and dressing children
Carrying and lifting children
Gardening and Planting
Ask respondents to list other chores

35. Rank the **top 3 most difficult domestic task** by marking **"D"** and the **top 3 easiest domestic tasks** by marking 'X' besides elected tasks in the table below

(DDD= most difficult, XXX= easiest)

**Task**	**Physical stress Ranking**
Sweeping the floor	
Mopping the floor	
Cleaning the toilet and Bathroom	
Fetching and carrying water	
Fetching and carrying firewood	

Carrying food produce	
Washing dishes/kitchen ware	
Manually washing clothes	
Food preparation before cooking	
Cooking	
Bathing and dressing children	
Carrying and lifting children	
Gardening and Planting	
Other tasks specified by respondent ____________	

Question 34: Posture and movement

36. What work posture do you typically assume when doing any of the following each day? **(Choose the chore and position by marking "X" in the table and indicate how many hours/minutes per day you do chores in these positions).**

(Show respondents these positions and explain repetitive movement as performing the same wrist/hand movement many times in a minute (e.g. washing, cutting, grinding)

Chores	Sitting	Walking	Standing without walking	Back bent forward	Back Bending sideways/twisting	Neck bent and rotated	Squatting/ kneeling	Arms lifted above shoulders	Repetitive movement of the wrist and fingers (>10 mins)
Sweeping									
Moping the floor									

Washing dishes	
Washing clothes	
Carrying children	
Bathing children	
Dressing children	
Fetching water from well	
Carrying water	
Pounding food produce	
Grinding food produce manually	
Cooking food	
Other chores	

Question 37 to 41: Lifting of children and other objects

37. Do you **carry children** while performing household tasks?

☐Yes

☐No

☐Not applicable

38. If yes, how frequent do you carry children while performing household tasks

☐Rarely (once in a while)

☐Occasionally (some of the time)

☐Frequently (almost all the time)

☐Always (every time)

39. How do you typically carry children while performing other household tasks?

☐ On the Back

☐ On the Hip

☐ On the arm

☐ Others___________(please specify)

40. If yes, how old is/are the child or children you carry? ________ (age of child)

41.

42. On the average, how many times per workday do you lift, carry or transfer manually loads of different weight listed below?

☐ 0-5kg _______times

☐ 6-10kg__________times

☐ 16-25kg _________times

☐ More than 25kg_________times

☐ Others_____________(please specify)

Question 43 to 45: Support from family members

43. Do you ask any of your family member assistance with domestic tasks when you feel tired?

☐Yes

☐No

44. If yes how often do you ask for assistance from your family member with domestic tasks when you feel tired?

☐Never

☐Rarely

☐Sometimes/Occasionally

☐Always

45. To what extent does your children /spouse/any family member help in planning and performing housework?

☐Never participates (Helps with 0% of tasks)

☐Occasionally participates (helps with 1-24% of tasks)

☐Frequently participates (helps with 25-49% of tasks)

☐Always participates (helps with 50% or greater tasks)

Question 46: Stress appraisal and Time demand-control

46. Please mark an "X" below the response that most closely describes how much you agree with the statement.

**Statements**	**Strongly Disagree**	**Disagree**	**Neutral**	**Agree**	**Strongly agree**
I have sufficient time to complete the domestic tasks I need to do for the day.					

I have sufficient time for

hobbies or other activities that are meaningful to me.
I can effortlessly perform all my chores without assistance

Taking care of the home is not stressful.

I feel physically drained after completing the domestic tasks for the day.

When I am too tired or exhausted, I do not do my house chores

I enjoy doing domestic work

I am comfortable with performing house chores every day.

Doing household tasks requires a lot of physical effort.
Caring for children requires a lot of physical energy.
